# *Ex vivo* and *In vitro* antiplasmodial activities of approved drugs predicted to have antimalarial activities using chemogenomics and drug repositioning approach

**DOI:** 10.1016/j.heliyon.2023.e18863

**Published:** 2023-08-01

**Authors:** Douglas O. Ochora, Reagan M. Mogire, Rael J. Masai, Redemptah A. Yeda, Edwin W. Mwakio, Joseph G. Amwoma, Dancan M. Wakoli, Abiy Yenesew, Hoseah M. Akala

**Affiliations:** a Department of Biological Sciences, School of Pure and Applied Sciences, Kisii University, P.O. Box 408-40200, Kisii, Kenya; b DSI/NWU, Preclinical Drug Development Platform, Faculty of Health Sciences, North-West University, Private Bag X6001, 2520, Potchefstroom, South Africa; c United States Army Medical Research Directorate-Africa (USAMRD-A), Kenya Medical Research Institute (KEMRI)—Walter Reed Project, P.O. Box 54-40100, Kisumu, Kenya; d Kenya Medical Research Institute (KEMRI) – Kemri-Wellcome Trust Research Programme, P.O. Box 230-80108, Kilifi, Kenya; e Department of Biological Sciences, University of Embu P. O. Box 6-60100, Embu, Kenya; f Department of Biochemistry and Molecular Biology, Egerton University, P.O. Box 536-20115, Egerton-Njoro, Kenya; g Department of Chemistry, University of Nairobi, P.O. Box 30197-00100, Nairobi, Kenya

**Keywords:** Antiplasmodial, Chemogenomics, Drug reportioning, Irinotecan, Epirubicin, *Ex vivo*, *Plasmodium falciparum*

## Abstract

High malaria mortality coupled with increased emergence of resistant multi-drug resistant strains of *Plasmodium* parasite, warrants the development of new and effective antimalarial drugs. However, drug design and discovery are costly and time-consuming with many active antimalarial compounds failing to get approved due to safety reasons. To address these challenges, the current study aimed at testing the antiplasmodial activities of approved drugs that were predicted using a target-similarity approach. This approach is based on the fact that if an approved drug used to treat another disease targets a protein similar to *Plasmodium falciparum* protein, then the drug will have a comparable effect on *P. falciparum*. In a previous study, *in vitro* antiplasmodial activities of 10 approved drugs was reported of the total 28 approved drugs*.* In this study, six out of 18 drugs that were previously not tested, namely epirubicin, irinotecan, venlafaxine, palbociclib, pelitinib, and PD153035 were tested for antiplasmodial activity. The drug susceptibility *in vitro* assays against five *P. falciparum* reference strains (D6, 3D7, W2, DD2, and F32 ART) and *ex vivo* assays against fresh clinical isolates were done using the malaria SYBR Green I assay. Standard antimalarial drugs were included as controls. Epirubicin and irinotecan showed excellent antiplasmodial *ex vivo* activity against field isolates with mean IC_50_ values of 0.044 ± 0.033 μM and 0.085 ± 0.055 μM, respectively. Similar activity was observed against W2 strain where epirubicin had an IC_50_ value of 0.004 ± 0.0009 μM, palbociclib 0.056 ± 0.006 μM, and pelinitib 0.057 ± 0.013 μM*.* For the DD2 strain, epirubicin, irinotecan and PD 153035 displayed potent antiplasmodial activity (IC_50_ < 1 μM). Epirubicin and irinotecan showed potent antiplasmodial activities (IC_50_ < 1 μM) against DD2, D6, 3D7, and F32 ART strains and field isolates*.* This shows the potential use of these drugs as antimalarials. All the tested drugs showed antiplasmodial activities with IC_50_ values below 20 μM, which suggests that our target similarity-based strategy is successful at predicting antiplasmodial activity of compounds thereby circumventing challenges in antimalarial drug discovery.

## 1 Introduction

Worldwide, malaria is a major public health problem with 627,000 deaths out of 241 million cases reported in 2020 [1]. About 95% of the malaria cases reported in 2020 occurred in Sub-Saharan Africa [1]. Limited access to malaria treatment facilities, the emergence of insecticide-resistant vectors [2], and emerging drug-resistant parasites [3] have contributed to the high malaria prevalence. More recently, resistance has been reported to artemisinin-based combination therapy (ACT) in Southeast Asia, where antimalarial drug resistance is most prevalent [2], including resistance to artemether-lumefantrine, which is recommended by the World Health Organization as the first-line treatment for uncomplicated *P. falciparum* malaria [4]. The trend in drug resistance and parasite evolution necessitates the search for alternative antimalarial drugs with novel modes of action.

Search for antimalarial drugs in the past relied upon testing plants traditionally used as remedies for fever and malaria [5], leading to the discovery of successful antimalarials such as artemisinin from *Artemisia annua* L. (Asteraceae) and quinine from *Cinchona ledgeriana* Bern. Moens (Rubiaceae) [6]. Initial antimalarials have often served as scaffolds for newer regimens, minimizing the emergence of resistance. Other molecules such as flavonoids and quinolines have been modified to form analogs with potent antimalarial properties [7,8]. These methods are expensive, time-consuming and many molecules fail to progress to approval due to safety requirements [9]. This therefore calls for alternative methods for antimalarial drug discovery.

Computer-Aided Drug Discovery and Development has been used to reduce the cost, the duration and overcome safety challenges that are involved in traditional antimalarial drug discovery [10,11]. This includes the use of target similarity approach which is based on the principle that if a *Plasmodium* parasite protein is similar to a putative drug target protein, then the drug in question would have a similar effect on the protein of interest. Previous studies have used the same approach in repositioning drugs against *P*. *falciparum* apicoplast and *Schistosoma mansoni* [[12], [13], [14]]. We used a similar strategy to predict approved drugs with activity against *P. falciparum* [15], although the *in vitro* antiplasmodial activity of many of the predicted approved drugs remains unknown. Therefore, the current study focused on the antiplasmodial activities of approved drugs with unknown antimalarial activities.

As part of our ongoing studies of identifying antiplasmodial activities of approved drugs that target proteins similar to those present in *Plasmodium* proteome, this study describes *in vitro* and *ex vivo* antiplasmodial activities of six approved drugs that were predicted using a target similarity approach in a previous study [15]. The drugs reported in this study are already approved and therefore overcoming cost, time and safety challenges in antimalarial drug development and discovery. This is likely to therefore hasten discovery of alternative antimalarial drugs.

## 2 Materials and methods

### 2.1 Drugs used for antiplasmodial tests

This work is part of our ongoing study which predicted approved drug targets with antiplasmodial activity. This was done by searching approved drugs with known drug targets that were similar to *P*. *falciparum* proteins on three drug databases, namely DrugBank, Therapeutic Target Database (TTD), and STITCH 4.0. This led to the identification of 28 approved drugs with unknown antiplasmodial activity [15]. Antiplasmodial activities of six of the drugs that were not previously tested are hereby reported, including irinotecan, PD153035 HCL, venlafaxine, pelinitib, epirubicin and palbociclib. These test drug compounds were obtained from Pfizer and the University of Potsdam, Germany.

Chloroquine (CQ), mefloquine (MQ), artemether (ART), Artemisinin (ATM), lumefantrine (LU), dihydroartemisinin (DHA), piperaquine (PPQ) and quinine (QNN) were obtained from World Wide Antimalarial Resistance Network (WWARN) under the on-going External Quality Assurance Programme, Bangkok, Thailand [16] and were tested as reference standard drugs alongside the candidate drugs.

### 2.2 *In vitro* antiplasmodial tests

The following cells were obtained through Biodefense and Emerging Infectious Research Resources Repository (BEI Resources), National Institute of Allergy and Infectious Diseases (NIAID), National Institutes of Health (NIH): *P. falciparum*, strain D6, Magnetic Resonance Angiography (MRA) 285, strain DD2, MRA-150, donated by David Walliker, strain W2, MRA-157 donated by Dennis E. Kyle, strain 3D7, MRA-102, contributed by Daniel J. Carucci, while strain F32 ART (artemisinin-resistant) was a kind donation from the Institute of Pasteur. These strains exhibit the following phenotype; W2 (chloroquine-resistant, mefloquine sensitive and artemisinin sensitive Indochina), DD2 (chloroquine-resistant, mefloquine resistant), D6 (mefloquine-resistant, chloroquine-sensitive Sierra Leone I), 3D7 (chloroquine-sensitive, mefloquine sensitive) and F32 ART (artemisinin-resistant). The strains were revived from a liquid nitrogen freezer and maintained in continuous culture as previously described by Trager and Jensen 1976 [17,18] to attain a parasitemia of 3–8%.

Drugs were dissolved in 99.5% dimethylsulfoxide (DMSO) (Sigma-Aldrich) and then diluted in a complete Roswell Park Memorial Institute (RPMI 1640) cell culture medium. Drugs at serial dilutions of; mefloquine (0.488–250 ng/mL), chloroquine (1.953–1000 ng/mL) alongside test-approved drugs (24.414–2000 ng/mL) were generated on a 96-well plate with DMSO ≤0.0875% [19].

A modified non-radioactive malaria SYBR Green I assay technique [20] was used to determine the IC_50_s of the approved drugs with suspected antimalarial activities against the five laboratory-adapted strains of *P. falciparum* (W2, DD2, D6, 3D7, and F32 ART) alongside fresh clinical isolates. Briefly, the parasites with a dose range of reference and test drugs were then added to 96 well plates. The plates were incubated at 37 °C in a gas mixture of 5% CO_2_, 5% O_2_, and 90% N_2_ for 72 h. The assay was then terminated by adding lysis buffer containing SYBR Green I dye (1 × final concentration) and then incubated in the dark at room temperature for 24 h. Lysis buffer was prepared as earlier described by Akala et al., 2011 [21]. Relative fluorescence units (RFUs) of each well were used to determine the parasite growth inhibition by use of Tecan Genios Plus (Tecan US, Inc., Durham, NC) set at an excitation wavelength of 485 nm and emission wavelength of 535 nm, the number of flashes was set at 10, the gain set at 60 with and integration time set at 40 μs?

### 2.3 *Ex vivo* antiplasmodial tests

Fresh blood samples were collected from individuals presenting at Kisumu County Hospital and Kombewa Sub-County Hospital within Western Kenya as described by Akala et al., 2011 [21]. A written informed consent/assent was obtained from each individual before each blood draw. The samples were tested within 6 h of collection. Parasitemia was determined and the presence of *P. falciparum* was confirmed at the malaria drug resistance lab using microscopic techniques. Samples whose parasitemia exceeded 1% parasitemia were adjusted to 2% hematocrit and 1%. Erythrocytes, RPMI media, and parasites were added to 96 well plates coated with dose ranges of reference and test drugs. The plates were then incubated, the assay was terminated and plates read as described in section 2.2.

### 2.4 Data analysis

The RFUs readings were analyzed using Graph Pad Prism® 8.0 windows software (Graph Pad Software, San Diego, CA, USA) to calculate IC_50_s of the test drugs. More than three separate readings from each sample were presented as mean ± standard deviation (Mean IC_50_ ± SD). The single factor Analysis of Variance (ANOVA) was used to determine if there was a significant difference in the means of the test drugs across all *P. falciparum* strains and field isolates. The Turkey's post-hoc test was also done to determine the difference between the drugs and Dunnett's post-hoc test was done to further check if the means of the approved drugs were different from the control groups, chloroquine (CQ) and mefloquine (MQ). Data was analyzed using GraphPad Prism® 8.0 windows software (GraphPad Software, San Diego, CA, USA).

### Ethical approval

The current study was approved by the Scientific and Ethics Review Unit (SERU) of Kenya Medical Research Institute (KEMRI), Nairobi, Kenya, and Walter Reed Army Institute of Research (WRAIR) Institutional Review Board, Silver Spring, MD, under protocols KEMRI/SERU/CCR/0018/3126, WRAIR#2257 for antiplasmodial activities of compound libraries for malaria drug discovery and development, KEMRI-SSC# 3628, WRAIR# 2454 for use of field isolates and KEMRI-SSC# 11,910, WRAIR# 1384 for use of human blood cells.

## 3 Results

### 3.1 Antiplasmodial activities

Table 1 and Table 2 below summarizes the IC_50_s for all the drugs tested. Mean IC_50_s across different strains of *P. falciparum* and field isolates is also reported in Table 1. There was no significant difference (*P* = 0.337) in the mean IC_50_s of the tested drugs across different strains of *P. falciparum* and the field isolates. Similarly, when the test drugs were compared with the control drugs (CQ and MQ), there was no significant difference.

**Table 1:** Antiplasmodial activities of approved drugs in mean IC_50_s (μM ± STDEV) against *P. falciparum* strains (*P* = 0.337).

Drugs'	W2 strain (n = 6)	DD2 strain (n = 6)	D6 strain (n = 6)	3D7 strain (n = 6)	F32 ART strain (n = 6)	Field isolates *ex vivo* (n = 6)	Mean IC_50_ of drugs across *P. falciparum* strains and field isolates
Epirubicin	0.004 ± 0.0009	0.155 ± 0.0759	0.194 ± 0.028	0.155 ± 0.022	0.393 ± 0.157	0.044 ± 0.033	0.158^a^^a^^b^^a^^d^^a^^e^^a^^f^^a^^g^^a^^h^^a^
Irinotecan	1.928 ± 6.023	0.238 ± 0.198	0.243 ± 0.203	0.278 ± 0.327	0.038 ± 0.024	0.085 ± 0.055	0.468^a^^a^^b^^a^^c^^a^^e^^a^^f^^a^^g^^a^^h^^a^
Pelitinib	0.057 ± 0.013	2.005 ± 0.613	3.026 ± 0.617	1.591 ± 0.396	1.63 ± 0.222	0.212 ± 0.098	1.42^a^^a^^b^^a^^c^^a^^d^^a^^f^^a^^g^^a^^h^^a^
Venlafaxine	1.497 ± 0.032	2.948 ± 0.514	3.193 ± 0.365	1.31 ± 0.565	2.662 ± 17.861	0.397 ± 0.171	5.995^a^^a^^b^^a^^c^^a^^d^^a^^e^^a^^g^^a^^h^^a^
Palbociclib	0.056 ± 0.006	NS	4.091 ± 1.621	5.776 ± 2.126	NS	0.311 ± 0.102	2.556^a^^a^^b^^a^^c^^a^^d^^a^^e^^a^^f^^a^^h^^a^
PD 153035	1.84 ± 2.633	0.194 ± 0.117	1.11 ± 0.601	0.692 ± 0.507	1.361 ± 1.307	0.235 ± 0.047	3.665^a^^a^^b^^a^^c^^a^^d^^a^^e^^a^^f^^a^^g^^a^
Chloroquine	0.175 ± 0.086	4.694 ± 0.686	0.006 ± 0.003	0.457 ± 0.068	0.02 ± 0.006	0.215 ± 0.091	0.928^b^^a^^c^^a^^d^^a^^e^^a^^f^^a^^g^^a^^h^^a^
Mefloquine	0.0003 ± 0.0002	0.022 ± 0.007	0.002 ± 0.001	0.0025 ± 0.009	0.005 ± 0.003	0.058 ± 0.016	0.015^a^^a^^c^^a^^d^^a^^e^^a^^f^^a^^g^^a^^h^^a^

W2: chloroquine-resistant, mefloquine sensitive; DD2: chloroquine-resistant, mefloquine resistant; D6: mefloquine-resistant, chloroquine-sensitive; 3D7: chloroquine-sensitive, mefloquine sensitive; F32 ART: artemisinin-resistant, strains of *P. falciparum*; STDEV: standard deviation; NS: assay was not successful; values are 50% inhibition concentration means ± standard deviation in micro molar.

a *P* > 0.05, a = compared with CQ, b = compared with MQ, c = compared with epirubicin, d = compared with irinotecan, e = compared with pelinitib, f = venlafaxine, g = compared with palbociclib, h = compared with PD 153035.

**Table 2:** Antiplasmodial activities of other standard antimalarial drugs against freshly collected clinical isolates in mean IC_50_s (μM ± STDEV).

Antimalarial drugs	Field isolates *ex vivo* from two nearby hospitals (n=4), *P* value = 0.005	
	HK1	HK2
Quinine	0.105±1.12	0.065±14.69
Artemisinin	0.0152±0.01	0.014±1.33
Dihydroartemisinin	0.003±0.21	0.007±1.52
Piperaquine	0.07±3.78	0.068±6.47
Artemether	0.008±0.46	0.004±0.61
Lumefantrine	NS	0.055±28.51

NS: assay was not successful; values at 50% inhibition concentration means ± standard deviation in micro molar; STEV: standard deviation; HK1: field samples from hospital 1; HK2 field samples from hospital

Epirubicin was the most active drug against all tested *P. falciparum* strains except for the F32 ART malaria parasite strain. Epirubicin (IC_50_ = 0.004 μM), pelinitib (IC_50_ = 0.057) and palbociclib (IC_50_ = 0.057 μM) showed excellent antiplasmodial activity (IC_50_ < 1 μM) against W2 *P. falciparum* strain. PD 153035 had the least activity with an IC_50_ value of 1.84 μM against the W2 strain (Table 1).

The test drugs; PD 15393035, epirubicin, and irinotecan depicted potent antiplasmodial activities against the DD2 strain with IC_50_ values of 0.194 μM, 0.155 μM, and 0.238 μM, respectively. A similar trend of activity was observed with PD 15393035, epirubicin and irinotecan, against 3D7 with IC_50_ values of 0.692 μM, 0.155 μM, and 0.278 μM, respectively. Palbociclib showed good antiplasmodial activity with an IC_50_ value of 5.776 μM against the 3D7 strain, which was the least active of all tested drugs (Table 1). Conversely, both pelinitib and venlafaxine showed antiplasmodial activities with IC_50_ values of less than 3 μM (Table 1). Excellent antiplasmodial activities were also observed when epirubicin and irinotecan were tested against D6 and F32 ART strains of *P. falciparum* with IC_50_ values of less than 0.4 μM. The other four drugs (PD 15393035, palbociclib, pelinitib, and venlafaxine) showed antiplasmodial activities with IC_50_ values of more than 1 μM against the same strains (D6 and F32 ART). However, epirubicin and irinotecan showed excellent antiplasmodial activities with IC_50_ values of 0.393 μM and 0.038 μM respectively against the F32 ART artemisinin-resistant strain of *P. falciparum*. Epirubicin and irinotecan showed antiplasmodial activity with IC_50_ values of less than 0.4 μM when both were tested against 3D7 and F32 ART strains. Potent antiplasmodial activities were observed in all the tested drugs against field isolates *ex vivo* with IC_50_ values of less than 0.4 μM.

All drugs displayed a mean IC_50_ of less than 6 across all strains and field isolates with the highest mean activity expressed by epirubicin (mean IC_50_ = 0.158 μM) and irinotecan (mean IC_50_ = 0.468 μM) (Table 1). All drugs showed higher activity when tested against freshly collected field isolates *ex vivo* than when tested against cultured adapted strains of *P. falciparum in vitro.*

## 4 Discussion

### 4.1 Antiplasmodial activities

Antimalarial drug development and discovery from traditional methods such as the screening of plants for antimalarial activity takes a lot of time, it is costly, and some fail to get approval due to safety reasons [9]. This leaves a few compounds in the pipeline for the development as antimalarial drugs. The current study is on the antiplasmodial activities of six drugs approved for treatment of other diseases but with no report on their antimalarial activity. The results provide insight into the potential use of these approved drugs as antimalarials thereby overcoming some of the challenges in antimalarial drug discovery. Antiplasmodial activities of pure compounds of the approved drugs was defined as follows in this study: (i) IC_50_ >200 μM inactive; (ii) IC_50_ 100–200 μM low activity; (iii) IC_50_ of 20–100 μM moderate activity; (iv) IC_50_ of 1–20 μM good activity; and (v) IC_50_ of <1 μM excellent/potent [22].

The drugs displayed no significant difference (*P* = 0.337) in the terms of their antiplasmodial activities across different strains of *P. falciparum* and field isolates. Similarly, the Dunnet’s post-hoc test revealed that the approved drugs tested did not significantly differ (*P* > 0.9) in the means of the antiplasmodial activities from control drugs, except for venlafaxine (*P* = 0.293 compared with CQ and *P* = 0.156 compared with MQ) and PD 153035 (*P* = 0.625, compared with MQ). This indicates that the sample drugs' ability to clear *P. falciparum* parasites is comparable to reference antimalarial drugs. Therefore, the tested drugs generally demonstrate a high malaria parasite clearance across different strains of *P. falciparum* and freshly collected field isolates. The observed potent antiplasmodial activities (IC_50_ < 1 μM) of the test drugs; PD 15393035, epirubicin, and irinotecan against the DD2 strain and PD 15393035, epirubicin and irinotecan, against 3D7 strain is suggestive of their potential as candidate antimalaria drugs. These drugs exhibit high parasite clearance that is comparable to currently used antimalarial drugs. Epirubicin exhibited excellent antiplasmodial activity with a mean IC_50_ value of 0.158 μM which was better than that of CQ with a mean IC_50_ value of 0.928 μM across all strains of *P. falciparum* and field isolates. The antiplasmodial activity of epirubicin compares well with other studies [23], especially to Ferreira and colleagues’ study that used computational chemogenomics and drug repositioning [23], where epirubicin displayed potent *in vitro* antiplasmodial activity against three different strains of *P. falciparum*; 0.111 μM (3D7), 0.099 (DD2) and 0.069 (W2). Similarly, irinotecan exhibited a mean IC_50_ value of 0.468 μM when tested against different strains of *P. falciparum* and fresh field isolates. This value was significantly similar to that of CQ (*P*=0.292) and MQ (*P*=0.078). The observed similarity depicts that the drugs have similar therapeutic activity to standard antimalarial drugs and therefore the high inhibition activity against *P.* f*alciparum*. Similar antiplasmodial activity of approved drugs with unknown antimalarial activity has been observed previously [15].

The *ex vivo* antiplasmodial activities of the approved drugs with unknown antimalarial activities (Table 1) were slightly lower than the selected standard antimalarial drugs when tested against freshly collected field isolates (Table 2). There was no significant difference (*P* = 0.42) between the antiplasmodial activities of the standard antimalarial tested against fresh field isolates from the two hospitals (HK1 and HK2) (Table 2). Antiplasmodial activities of epirubicin and irinotecan against field isolates were comparable with most of the tested standard antimalarial drugs (Table 1 and Table 2). The observed antiplasmodial activities further indicate a potential use of these two drugs as antimalarials. Considering that the samples tested in this study are approved drugs, therefore they are considered safe. Moreover, additional bioactivity information of the drugs is known which can be related to the reported antiplasmodial activity in this study. These findings will play a key role in hastening the use of these drugs for the treatment of malaria.

### 4.2 Antiplasmodial activities’ relation to druggability index and consurf analysis

In addition to potent antiplasmodial activities, DNA topoisomerase II (the *P. falciparum* protein) which is similar to DNA topoisomerase 2-alpha (the putative protein target of epirubucin), the targeted protein has a high percentage of conserved amino acids of 61% according to the Consurf analysis (Table 3). This was the highest Consurf analysis percentage among all the *P. falciparum* proteins that are similar to the proteins of the drugs tested in this study. Protein–protein pairwise alignment was performed to determine conserved amino acids in a protein [23]. This identifies functional amino acid residues shared between *P. falciparum* proteins and its homologous putative drug targets. Since these amino acids are preserved over many generations, they are believed to play key structural and functional roles in these proteins. By searching on NCBI using a drug target as the query and the *P. falciparum* homologous target as a subject, protein pairs with > 80% query coverage progressed for Consurf server analysis [24].

**Table 3:** Details of approved drugs predicted to target *P*. *falciparum* proteins

Drugs	Putative target	Uniprot ID	*P. falciparum* protein	NCBI accession number of *P*. *falciparum* target	CONSURF results (Mogire et al., 2017)	Druggability of *P*. *falciparum* target (Mogire et al., 2017)	Approved drug use	Mode of action (inhibition)
Epirubicin	NA	P11388	DNA topoisomerase II	XP_001348490.1	61%	0.8	Breast cancer	DNA topoisomerase 2-alpha
Palbociclib	NA	P11802	MO15-related protein kinase	XP_001347426.1	54%	0.9	Breast cancer	CDK4/6
Irinotecan	ATP-binding cassette sub-family Gmember 2	Q9UNQ0	ABC transporter	XP_001348418.1	51%	0.5	Colorectal cancer	DNA topoisomerase I
Venlafaxine	NA	Q9UNQ0	ABC transporter	XP_001348418.1	51%	0.5	Antidepressant	Serotonin and neropinephrine reuptake
Pelitinib	NA	NA	NA	NA	NA	NA	Colorectal and lung cancer	Epidermal growth factor receptor (EGFR) Tyrosine kinase
PD 153035	NA	NA	NA	NA	NA	NA	Lung cancer	EGFR Tyrosine kinase

NA: Not available

Consurf web server uses algorithmic tools to identify functional regions in proteins with a score being calculated using the Bayesian method. Consurf analysis percentage is based on the criteria that; > 80% has high similarity, 50-79% as moderate, and < 50% as low similarity. This indicates that the protein predicted to be targeted by the drug has many amino acids that play a key role in the life of *P. falciparum*. Therefore, this is suggestive of the high potential of epirubicin as an antimalarial drug.

The Druggability Index of DNA topoisomerase II (the *P. falciparum* protein) which is similar to the putative protein target of epirubicin was high at 0.8. Druggability index is the ability of a putative target to be therapeutically modulated by drug medication. This shows a likelihood of the *P. falciparum* protein being modulated by epirubicin drug molecule [25]. The most druggable protein has a score of 1.0 and the least druggable has a score of 0 [26]. This shows that the *P. falciparum* protein DNA topoisomerase II which has a Druggability Index of 0.9, is highly druggable and is the same target of epirubicin (Table 3). This explains the high antiplasmodial activity of epirubicin against *P. falciparum* strains and field isolates as reported in this study. The ABC transporter had the lowest Druggability Index of 51% which is considered to be moderate.

### 4.3 Limitations of the Study

The study could perform *in vivo* antiplasmodial assays using animal models due to financial constraints. This could give a better indication of the clinical use of the drugs as antimalarials since animals are exposed to factors such as diet and the natural environment which is likely to affect the pharmacokinetic and pharmacodynamics activities of the drugs.

## 5 Conclusion

Most drugs in this study showed excellent antiplasmodial activities that was comparable (*P* > 0.05) to standard antimalarial drugs (MQ and CQ) of which, epirubicin displayed the most potent antiplasmodial activity with the highest percentage in the Consurf analysis among the six drugs tested, along with high druggability index. Similarly, irinotecan displayed potent antiplasmodial activity with moderate Consurf percentage and moderate druggability index. Therefore, the two drugs have a high potential use for treatment of *P. falciparum* malaria which is the most lethal. Furthermore, these findings validate the use of a combination of target similarity approaches to predict approved drugs and *ex vivo* and *in vitro* assays to determine their antiplasmodial activities in antimalarial drug discovery. This will circumvent challenges such as safety, cost and time that hinder or delay approval of possible antimalarial drugs from reaching the market. Future *in vivo* studies should be conducted to confirm the antiplasmodial activity of these drugs and their use in the treatment of malaria using animal models.

## Author contribution statement

Douglas O. Ochora: Conceived and designed the experiments; Performed the experiments; Analyzed and interpreted the data; Wrote the paper.

Reagan M. Mogire: Hoseah M. Akala: Conceived and designed the experiments; Performed the experiments; Analyzed and interpreted the data; Contributed reagents, materials, analysis tools or data; Wrote the paper.

Rael J. Masai: Analyzed and interpreted the data; Wrote the paper.

Redemptah A. Yeda: Edwin W. Mwakio: Performed the experiments; Analyzed and interpreted the data.

Joseph G. Amwoma: Duncan M. Wakoli: Abiy Yenesew: Performed the experiments; Analyzed and interpreted the data; Wrote the paper.

## Data availability statement

Data included in article/supp. Material/referenced in article.

## Additional information

Supplementary content related to this article has been publish online at [URL].

## Declaration of Competing Interest

The authors declare that they have no known competing financial interests or personal relationships that could have appeared to influence the work reported in this paper.

## Appendix A Supplementary data

mmc1 mmc1
