# Supplementary material for: Ex vivo and In vitro antiplasmodial activities of approved drugs predicted to have antimalarial activities using chemogenomics and drug repositioning approach
Source: Heliyon. 2023 Aug 1;9(8):e18863. doi: 10.1016/j.heliyon.2023.e18863 (PMC10424068; doi:10.1016/j.heliyon.2023.e18863)

1. **SELECTED ANTIPLASMODIAL ACTIVITY DOSE RESPONSE CURVES**

**Figure S1:** Antiplasmodial activity dose response curves of tested drugs against D6 and DD2 *P. falciparum* clone.

**Figure S2:** Antiplasmodial activity dose response curves of tested drugs against W2 and 3D7 *P. falciparum* clone.

**Figure S3:** Antiplasmodial activity dose response curves of tested drugs against F32 ART *P. falciparum* clone and field isolates

**
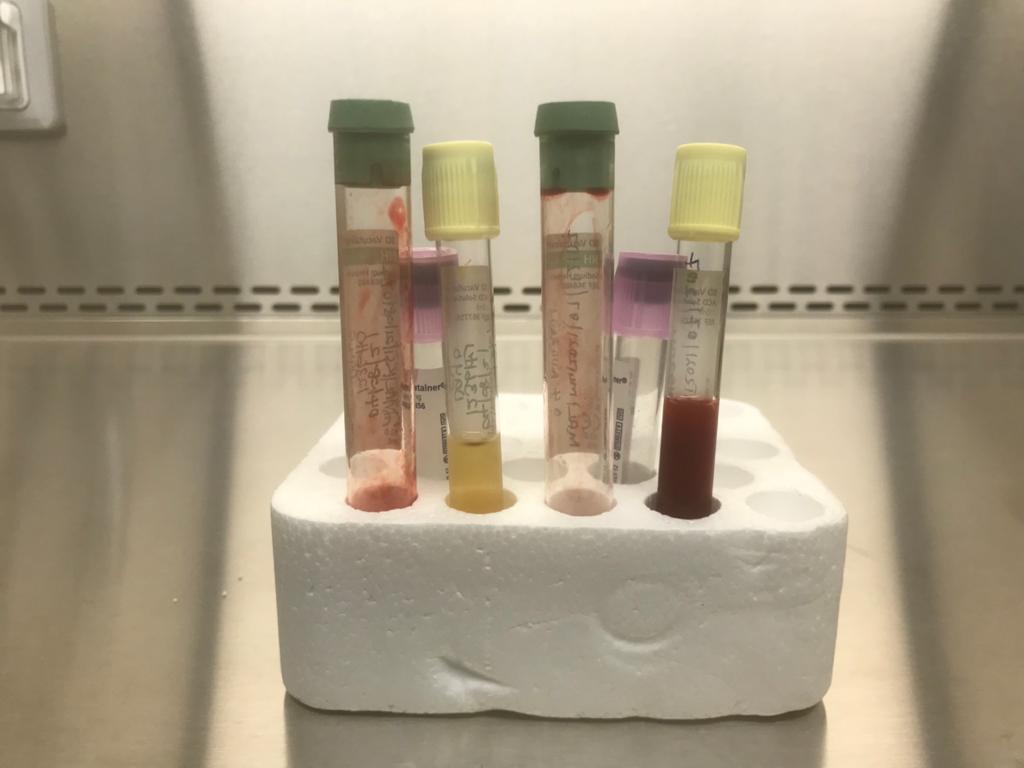
**

**Figure S4:** Blood samples receieved from nearby hospitals for immediate *ex vivo* assay.

**
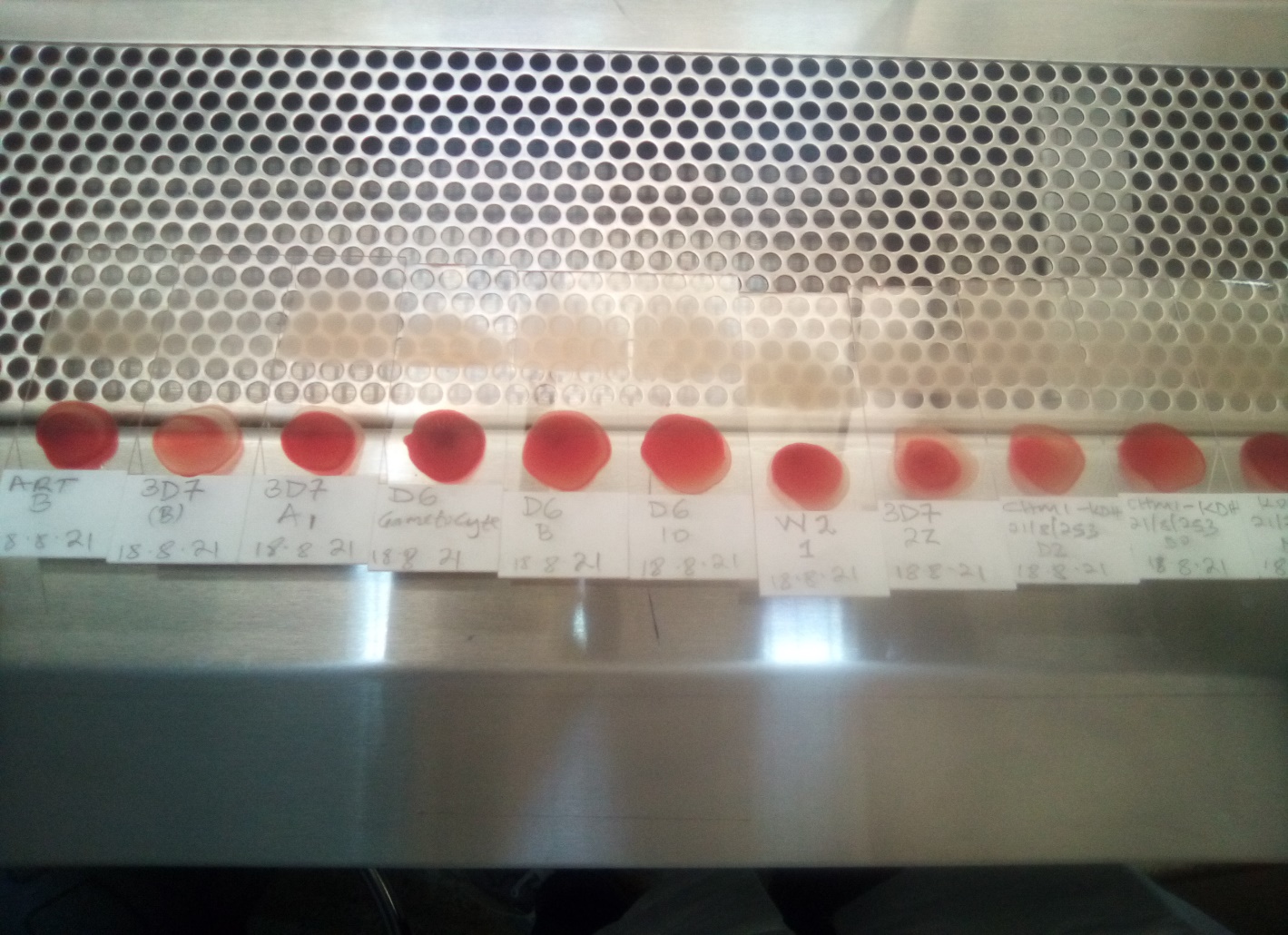
**

**Figure S5**: Slides for lab microscopic determination of parasitemia and confirmation of presence of *P. falciparum*

**
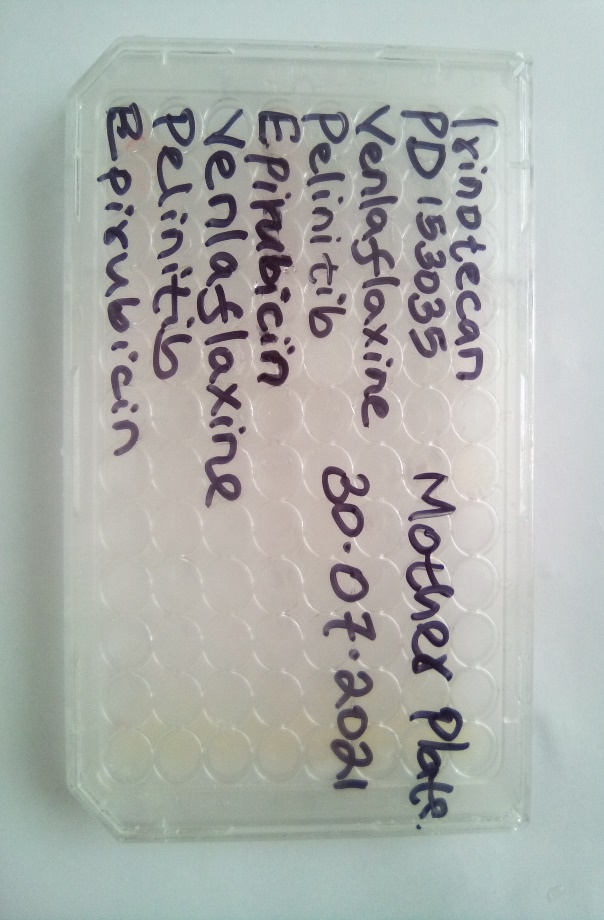
**

**Figure S6**: Drugs in 96-well plate after dissolving in 99.5 % dimethylsulfoxide (DMSO)

**
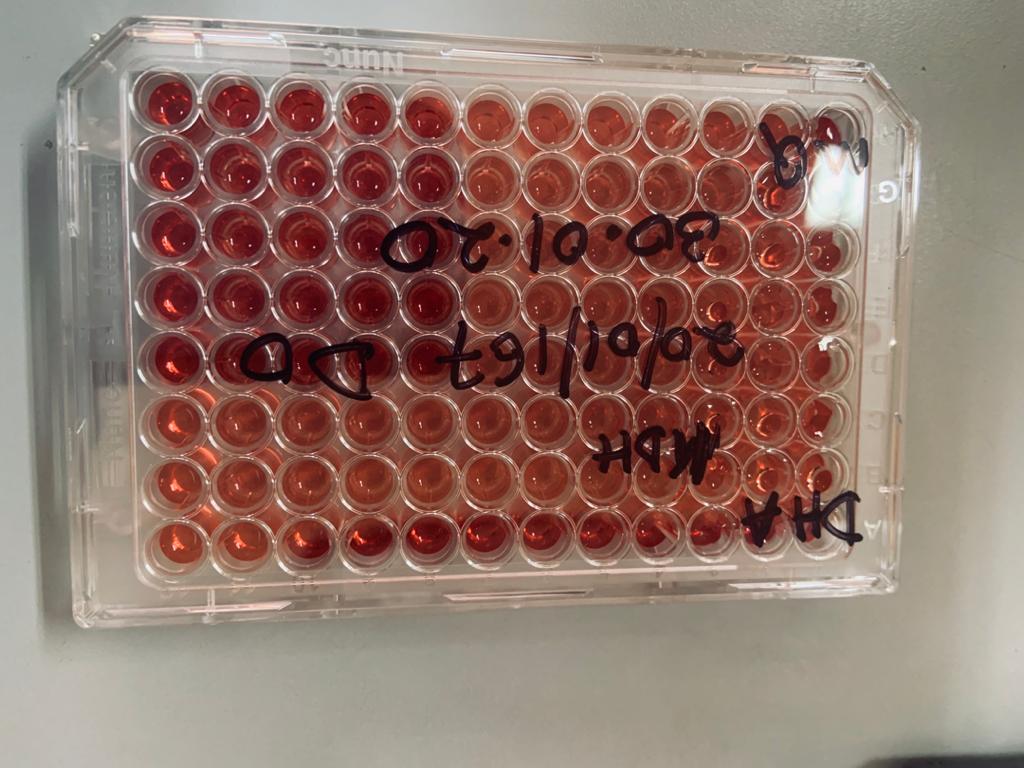
**

**Figure S7:** Red blood cells, drugs, *P. faciparum* strains and test media in 96-well plates.

1. **SELECTED RELATIVE FREQUENCY UNITS (RFUs) AND 50% INHIBITION CONCENTRATIONS (IC_50_s)**

**Table S1:** Relative fluorescence units IC_50_s for all the drugs tested against W2 *Plasmodium falciparum* clone.


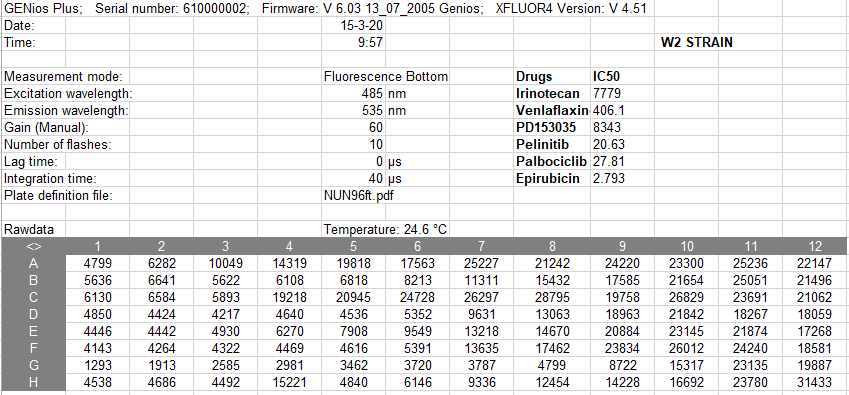


**Table S2:** Relative fluorescence units of tested drugs against KH2 field isolate *ex vivo.*


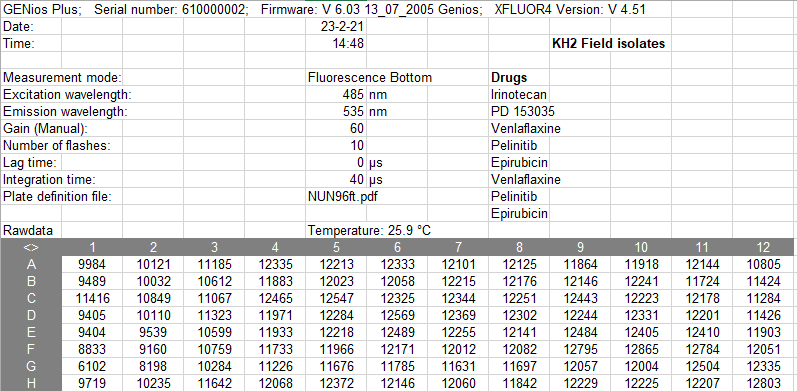

Supplement: mmc1 [file mmc1.docx]
